# Exploring the meaning and practice of self-care among palliative care nurses and doctors: a qualitative study

**DOI:** 10.1186/s12904-018-0318-0

**Published:** 2018-04-18

**Authors:** Jason Mills, Timothy Wand, Jennifer A. Fraser

**Affiliations:** 1 Institute of Health and Biomedical Innovation, Queensland University of Technology, Kelvin Grove, QLD Australia; 2 Susan Wakil School of Nursing and Midwifery, The University of Sydney, Camperdown, NSW Australia; 3 School of Nursing, Faculty of Health, Queensland University of Technology, Kelvin Grove, QLD 4061 Australia

**Keywords:** Palliative care, Self-care, Self-compassion, Positive emotions, Workforce

## Abstract

**Background:**

Self-care practice within the palliative care workforce is often discussed, yet seemingly under-researched. While palliative care professionals are required to implement and maintain effective self-care strategies, there appears little evidence to guide them. Moreover, there is an apparent need to clarify the meaning of self-care in palliative care practice. This paper reports qualitative findings within the context of a broader mixed-methods study. The aim of the present study was to explore the meaning and practice of self-care as described by palliative care nurses and doctors.

**Methods:**

A purposive sample of 24 palliative care nurses and doctors across Australia participated in semi-structured, in-depth interviews. Interviews were digitally recorded and transcribed prior to inductive qualitative content analysis, supported by QSR NVivo data management software.

**Results:**

Three overarching themes emerged from the analysis: (1) *A proactive and holistic approach to promoting personal health and wellbeing to support professional care of others*; (2) *Personalised self-care strategies within professional and non-professional contexts;* and (3) *Barriers and enablers to self-care practice.*

**Conclusions:**

The findings of this study provide a detailed account of the context and complexity of effective self-care practice previously lacking in the literature. Self-care is a proactive, holistic, and personalised approach to the promotion of health and wellbeing through a variety of strategies, in both personal and professional settings, to enhance capacity for compassionate care of patients and their families. This research adds an important qualitative perspective and serves to advance knowledge of both the context and effective practice of self-care in the palliative care workforce.

## Background

The concept of relentless self-care is well known to those in the field of palliative social work [1]. Interest in self-care is growing within the nursing and medical disciplines [2, 3], and its importance to all palliative care professionals is evident internationally through a suite of quality standards, core competencies, and practice standards in which self-care practice is mandated [4–10]. But what does self-care mean?

Self-care is broadly defined by Sherman [11] as ‘the self-initiated behaviour that people choose to incorporate to promote good health and general well-being’. Despite this health-promoting emphasis on good health and wellbeing, the palliative care literature focuses largely on coping strategies in the context of occupational stressors such as burnout or compassion fatigue [12]. Clearly, management of stress is very important; however, there are other important aspects of promoting good health and wellbeing that extend beyond the scope of coping with stress. In many cases there also appears to be conflation between the terms coping strategy and self-care strategy. Further confusion about the meaning of self-care was highlighted in an Australian survey of palliative care professionals [13]. Beyond academic definitions, there is a need to understand and articulate the meaning of self-care in a palliative care practice context. Given that some palliative care professionals have reported low levels of self-care ability, there is also an urgent need to explore barriers and enablers to self-care, and identify examples of effective self-care strategies used in practice. In reviewing the literature [12], significant gaps are apparent in the current evidence base for self-care practice and education.

To advance knowledge in these areas, this study aimed to explore the meaning and practice of self-care as described by palliative care nurses and doctors. Specifically, the following research questions were addressed:

1. What is the meaning of self-care, as described by palliative care nurses and doctors?
2. How do palliative care nurses and doctors describe effective self-care practice?

## Methods

### Research design

Given the nature of the research questions, a qualitative research design was employed [14]. An interview guide (see Table 1) was developed to address the study aim in consideration of gaps identified from the literature. The initial guide was refined in response to feedback received from a small group of palliative care professionals not involved in this study. Open questions were used to elicit deeper exploration of meaning and experience within a flexible yet focused discussion about participants’ self-care practice [15].

**Table 1:** Interview Guide

• In the context of palliative care practice, what does self-care mean to you? • From your experience, how would you describe effective self-care practice? • Tell me about the self-care strategies you find to be most effective • What supports your self-care practice? • What, if anything, gets in the way of your self-care practice?	

A purposive sample was recruited into this qualitative research from a pool of palliative care nurses and doctors who had completed a self-care survey as part of a broader mixed-methods study [13]. Consistent with the purpose of obtaining relevant and rich data from an appropriate source, eligible participants were nurses and doctors practising in Australia with palliative care as their main area of practice. Informed written consent was obtained from all participants through initial email contact prior to being interviewed via telephone. A total of 24 semi-structured, in-depth interviews were conducted over a six-month period in 2015, with recruitment ending once data saturation was reached. That is, when the collection of additional data served only to repeat existing rather than generate new content, as identified from the use of field notes and iterative analysis. The first author, an experienced qualitative researcher, conducted all interviews and recorded field notes to support a process of iterative analysis throughout the data collection period. The average duration of interviews was approximately 50 min, with audio content digitally recorded, transcribed verbatim, and de-identified. Gender-appropriate pseudonyms were randomly allocated to each respondent.

### Data analysis

Interview transcripts were initially read and re-read to make note of key words and phrases before importing them into QSR NVivo 11 data management software for open coding and qualitative content analysis. Qualitative content analysis is a widely used method for interpreting the content of textual data through a process of systematic classification, coding, and identification of patterns or themes [16]. As recommended by Graneheim and Lundman [17], a number of decisions were made to guide content analysis and ensure trustworthiness.

**Fig. 1:**
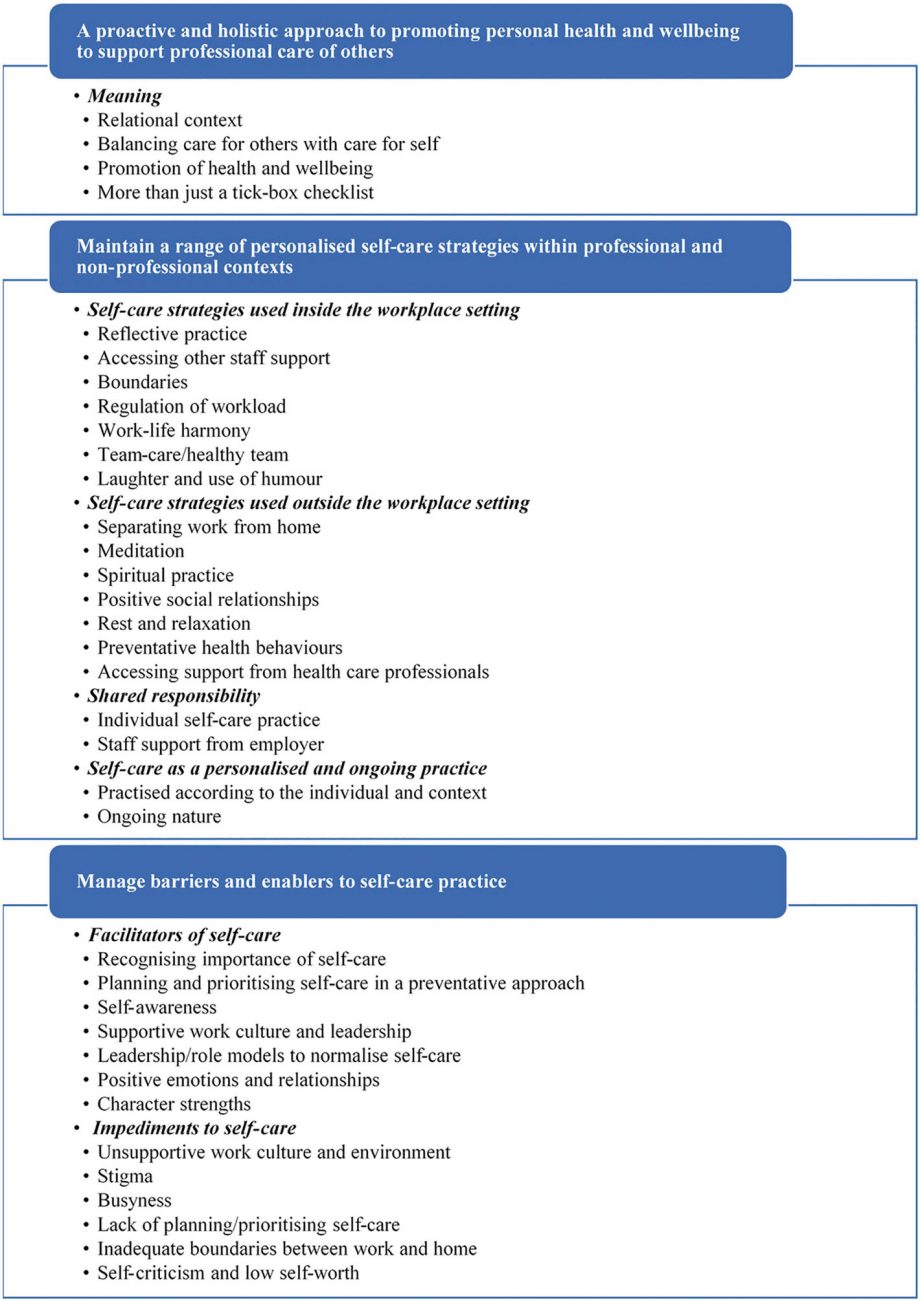
Thematic coding and category content

First, it was decided that a conventional approach to content analysis would be adopted [16]. That is to say, the analysis was inductive and focused on latent content, or words and sentences that required an interpretation of underlying meaning. Second, whole participant interviews were discerned to be the most appropriate unit of analysis in terms of providing a context for meaning units during the analytical process [17]. Finally, it was decided that these meaning units would comprise interrelated words, sentences and paragraphs from interview transcripts. In this way, interview data were analysed inductively through the generation of codes, grouping and collapsing of codes into common categories, and subsequent abstraction to identify overall themes that represent the raw data in an aggregated form [18, 19]. Figure 1 outlines the thematic coding and category content generated from the content analysis.

### Sample

The sample of 24 participants comprised 12 nurses and 12 doctors working in community, inpatient, or consult palliative care services located in both metropolitan and regional/rural settings across six of the eight States and Territories in Australia. These were clinical nurse specialists, nurse educators, clinical nurse consultants, nurse practitioners, nurse unit managers, senior medical officers, consultant physicians, and heads of department. They had an average of 15 years’ experience working in either adult, aged, or paediatric palliative care settings. Most were female, aged between 40 and 49, and worked in full-time roles. See Table 2 for detailed participant demographics.

**Table 2:** Participant Demographics and Professional Characteristics (*N* = 24)

Demographic	n (%)
Gender	
Female	15 (63)
Male	9 (37)
Age Group	
30–39 years	4 (17)
40–49 years	11 (46)
50–59 years	7 (29)
≥ 60 years	2 (8)
Population Focus	
Adult Palliative Care	19 (79)
Paediatric Palliative Care	2 (8)
Aged Palliative Care	3 (13)
Work Status	
Full-time	14 (58)
Part-time	10 (42)
Years Worked in Palliative Care	
1–5 years	1 (4)
6–10 years	3 (13)
11–15 years	12 (50)
≥ 16 years	8 (33)

## Results

**Fig. 2:**
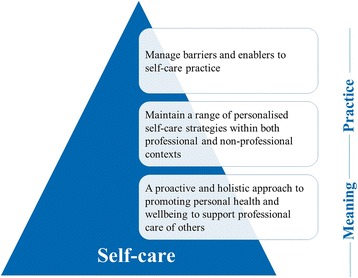
The meaning and practice of self-care

Three overarching themes emerged from the analysis in relation to the meaning and practice of self-care: (1) *A proactive and holistic approach to promoting personal health and wellbeing to support professional care of others*; (2) *Personalised self-care strategies within professional and non-professional contexts*; and (3) *Barriers and enablers to self-care practice.* Figure 2 illustrates the application of these as the meaning and practice of self-care*.* Thematic data from these themes are reported in Tables 3, 4, and 5, in the form of participant quotations to ensure trustworthiness [20].

**Table 3:** Theme 1

A proactive and holistic approach to promoting personal health and wellbeing to support professional care of others	
Prudence	*Through self-care, what we are doing is developing a relationship with ourselves – which actually supports us in developing relationships with everybody else.*
Gwendolen	*Self-care - it’s looking after me to look after patients, so to speak; if I’m not of a good healthy physical state or emotional state, I’m hardly likely to be able to support someone.*
Darrell	*You can’t look after dying patients without looking after yourself, really, can you? And do a good job of that, in a compassionate way?*
Felicity	*Balancing care for yourself and others is essential.*
Patrick	*It’s part of a holistic approach… if you’re not caring for yourself then you’re less able to care for others.*
Merilyn	*[It’s about] maintaining a good balance between body and mind… being able to stay fit and healthy.*
Winston	*You look after your own health so that you can deliver patient-centred care.*
Prudence	*That’s what self-care is; it’s a way of living, it’s a way of living every moment.*
Mason	*The thing is, [self-care] is not a tick-box commodity.*

**Table 4:** Theme 2: Personalised self-care strategies within professional and nonprofessional contexts

Philis	*It’s really not formulaic; it’s really quite individual, and so everybody has to find their own way of doing it.*
Carmel	*You not only need self-care strategies in the workplace - but also in your personal life.*
Felicity	*I do try and exercise a reasonable amount and I try and get to bed on time because I have to get up at a reasonable time… and diet’s important.*
Gwendolen	*I regularly exercise, do yoga and have a regular massage as well.*
Abbie	*[Having] a bath; it’s almost like I’m washing the hospital off me.*
Larissa	*I have an extremely supportive, very good husband and I have an extremely good network of friends, so… spending time with family and friends.*
Doreen	*Maintaining relationships with family; making sure I’m spending a reasonable amount of time with my children makes me feel that all is right in the world.*
Abbie	*Meditate for half an hour a day; that’s all I actually need to do to function well - I’m great at work, I’m calm with [son]. But if I don’t do that, then I get irritable [and] I don’t have as much to give at work. With just half an hour of meditation a day as my top priority for the day, I’m just better all round.*
Lucas	*I’m quite involved in my Church, and faith is actually a big anchor [that keeps me grounded].*
Reece	*…my spiritual practice which, for me, is a very reliable tool; Buddhist practices… to do with strengthening my connection with compassion or loving kindness for self and others.*
Deanna	*Work-life balance is really important.*
Sandra	*There’s no such thing as work-life balance, it’s rubbish.*
Cathi	*If it’s after five o’clock: (a) I won’t be [at work]; and (b) my diary will be sitting on my desk with my mobile phone on it turned off, with my name tag sitting on it. My computer will be off. [It’s] about making certain that work is at work, so I don’t take my mobile phone home. I don’t take my diary home [to follow up on things]; no, sorry that’s work, and it will wait… work stays at work.*
Felicity	*I take the train… I can only arrive at a certain time and leave at a certain time – those boundaries are actually very helpful. I’ve never been very good at placing boundaries, so I actually have to do this physical boundary of ‘Right, the train’s leaving, and I have to go’ - and that’s worked quite well.*
Deanna	*My de-escalating time is driving home and when I walk in the door at home, work stays at work… and I find that something to be really important to me actually – that the two don’t intermix.*
Winston	*It’s not sustainable to give out more than you really can on an ongoing basis… absolutely [regulating work demands is important].*
Cathi	*I take regular holidays. I’m not somebody who’s got an annual leave balance; I always take my meal breaks, take my days off, and sick leave when ill.*
Larissa	*I chose [to work] part-time.*
Scott	*It’s very difficult to do self-care without [team] support, and so you support each other in doing self-care at work, definitely.*
Doreen	*A mindful activity, just grounding yourself …this conscious thing of ‘Okay, what can I see? What can I feel? What am I touching?*
Carmel	*Supervision [provides] a safe and guided reflective space that allows you to talk about your practice; to think about what is meaningful to you, about a time, something you did, something you’ve been experiencing recently… allow yourself to really drill into - not just the story - but how did it make you feel… how did you behave… what would you change?*
Darrell	*[informal debriefing] is a sign of a healthy team because that’s… self-initiated, as opposed to organised or imposed.*
Deanna	*It’s Friday, I’m tired. A lot has gone on, and I’m giving a handover. I get half-way through the ward and then I start wrapping up. And they go, ‘What are you doing? There’s still the other half of the ward to go yet’, and I’m like, ‘Oh, damn’! So, being able to [make a mistake] and be able to laugh about it was important. Being kind and being compassionate about that. Being able to accept that you are human.*
Abbie	*We’ve all got a very black sense of humour, so it works really well.*

**Table 5:** Theme 3: Barriers and enablers to self-care practice

Cathi	*There’s too many patients and you can’t get enough done… busyness contributes to poor self-care because you actually don’t stop to go ‘How has this affected me? What can I do? What do I need to make me ‘okay’ about it?’*
Patrick	*There is an expectation that people won’t take holidays, but how are people supposed to recharge so they can keep working?*
Scott	*There’s this… culture sometimes where you just sort of ‘soldier on’ and do what’s expected – take work home.*
Merilyn	*It just follows you home and it can really impact on your home life and your health, because you’re just… stressing about things*
Gordon	*The biggest hindrance to self-care is organisational culture.*
Larissa	*There’s a big culture shift that needs to ha in order for people to be able to look after themselves properly*.
Winston	*There is still a lot of stigma around having feelings or accepting feeling or being vulnerable… we do see confronting things but there is still that superhero, you know, not letting it affect you.*
Peggie	*People are considered to be selfish if they do something for themselves… you know, if you take a day off because you’re on a mental health day people think ‘Oh, she’s so selfish because she’s let her team down’*
Prudence	*Lack of self-worth and self-value is a bit of an issue… I can see that in how my colleagues - how people treat themselves, and that’s not a judgment - it’s an observation coming from someone doing pretty much the same kind of thing.*
Sandra	*Self-care always get shoved down to the bottom… that self-worth thing of… you know, something else always being much more important.*
Philis	*I certainly used to be quite critical about myself… which isn’t a particularly helpful thing to do really… [most of us] just beat ourselves up emotionally and physically… and then eventually work out why you can’t keep doing that for the rest of your life.*
Carmel	*Self-care goes down the toilet when it’s random… there’s no effective random self-care.*
Peggie	*I’m very conscious of [self-care] because I’ve been a in bad spot before with palliative care… I really didn’t cope very well, so I’m [now] highly vigilant about self-care*.
Gordon	*I was ‘young and bullet-proof’… and I found [that] to be a fairly unpleasant experience; ‘young and bullet-proof’ didn’t work very well. But it took me about seven years to work that out, and I became significantly burnt out… So, having burnt out… taken time off, and readjusted… I’m now very conscious of how important self-care is.*
Abbie	*When I do [prioritise self-care] I’m calm and I’m more compassionate.*
Winston	*Preventative maintenance… Yeah, well it is [like having a regular check-up and a tune-up]*
Reece	*I have written self-care plans for myself… but I don’t approach that in a sense of, you know, at six months – ‘now I need to redo my self-care plan’. An ongoing planning process is the critical element rather than just the piece of paper.*
Carmel	*Leadership. It’s got to come from the top. You can’t have someone at the top who thinks that people who need to go for counselling are ‘poor little things’. Seriously, it’s not going to work*
Kaleb	*Having a reasonable degree of self-awareness is hugely important [for effective self-care], particularly in our line of work.*
Cathi	*Finding the positive in situations… also taking account of things that have negatively impacted me [but still finding] something positive.*
Doreen	*…intentionally choose how I want to be each morning, and how I want to leave work, and respond to events; having a mindset of gratitude.*
Reece	*Practising self-compassion is a really important enabler – without that I’m not really sure how authentic my self-care would be.*
Gordon	*Being realistic about your limitations is central to self-care… self-care involves being honest about a whole host of things, and it’s primarily being honest with yourself - and being prepared to take that up with other people where you need to - but it’s about being honest with yourself in relation to your limitations.*
Lucas	*Contemplating my own mortality is very important in terms of self-care. I have to have confronted that; there but for the Grace of God go I… and this could be me [dying]… Really puts things into perspective and helps you to live and enjoy your life to the full.*

### Theme 1: A proactive and holistic approach to promoting personal health and wellbeing to support professional care of others

The meaning of self-care was described in terms of its positive relational context with self and others. Although self-care was focused primarily on individual needs, it was informed by the broader clinical context of capacity to engage in positive and therapeutic relationships to provide patient care. Self-care meant fulfilling a fundamental part of palliative care practice, with one participant commenting that self-care *is intrinsic to the work itself.*

Self-care also meant balancing care for others with care for self, with the promotion of personal health and wellbeing central to its meaning. Self-care was described as a conscious and deliberate practice that meant much more than just a ‘tick-box’ checklist to be completed within a set of allocated tasks.

### Theme 2: Personalised self-care strategies within professional and non-professional contexts

Effective self-care practice was described as a personalised and ongoing endeavour. In the words of one participant, *it’s a constant work in progress*. Participant descriptions of effective self-care practices were consistently characterised by a variety of self-care strategies that were maintained both within, and external to, workplace settings.

#### Self-care in personal settings

Effective self-care strategies used outside of the workplace settings included a range of health behaviours, meditation and spiritual practice. A healthy diet, adequate sleep, and moderation of alcohol intake were considered important. In addition to exercising for fitness, other physical activities such as yoga and massage were found to be effective self-care strategies. Rest and relaxation at home in a bath were described as effective self-care strategies when feeling overwhelmed or needing to wash away (metaphorically) thoughts of the workplace. Socialising and maintaining positive relationships with friends and family was both supportive and meaningful. Meditation practice was also an effective self-care used within both personal and professional contexts. A variety of meditation practices were used by participants, including loving kindness meditation. Spiritual practice was also considered an effective self-care strategy.

Finding harmony between personal and professional roles was consistently described as an effective self-care strategy. Some described this harmony in terms of work-life balance. Interestingly, others found the concept of work-life balance to be problematic in practice. Given the elusive nature and individual context of what constitutes a balance between work and life, most considered it more important to acknowledge that different life-domains require varying degrees of attention at any given time; and that finding individual harmony between personal and professional roles was a key strategy towards flourishing in life. One participant explained:

*It’s never like you’ve got this nice balance - where work finishes, then you’ve got an hour to sort of wind down before the rest of life begins… [it’s more about] just trying to keep all the different areas of life flourishing* (Darrell).

Establishing and maintaining boundaries between home and the workplace was considered an effective self-care strategy. Some boundaries involved commuting to the workplace via modes of transport that prevented over-working, while for others the commute time itself constituted a process of unwinding from work so as to separate from it when arriving home.

#### Self-care in workplace settings

Boundaries were also relevant to effective self-care within the workplace. Awareness of boundaries in this context was supportive in terms of not over-working due to resource limitations, whilst also ensuring clarity of expectations for multiple stakeholders. In the words of one participant:

*The amount of resources [allocated to the] palliative care service - that actually creates a certain set of boundaries within which I can work - I’m not going to step over those boundaries; if they want additional work, they need to increase the resourcing… It’s about managing expectations around what I will do and what I won’t do - and being able to be very upfront in relation to that… with management… with staff that I work with, so [they] are aware of what we can do, and what we can’t; but more importantly, with patients and their families, so there’s a very clear set of expectations around what can reasonably be done for them* (Gordon).

Self-regulation of workload was important, but often difficult to achieve. It involved being assertive about one’s capacity in relation to workload and wellbeing. Taking meal breaks, taking recreation leave for regular holidays, and taking personal leave during illness were also considered effective self-care strategies. For some, choosing to work part-time was an effective self-care strategy that provided ongoing regulation of workload in relation to other competing demands.

Self-regulation as a self-care strategy was often supported by other members of the team. In this way, team-care was considered an aspect of effective self-care that contributed to a healthy team. One participant described an example of team-care in terms of checking in with colleagues about how they are feeling, as a reminder and invitation to attend to self-care.

Having a cohesive team was important and this contributed to a supportive working environment. Mindfulness exercises were an effective self-care strategy in the workplace, both in individual and group contexts. A sense of allowing oneself to be human, in the context of displaying emotion in the clinical setting, was also part of effective self-care practice.

Reflective practice, especially through participation in clinical supervision, was described by many as an effective self-care strategy, although formal supervision was not available in all participants’ workplace. Importantly, respect and confidentiality were important components for clinical supervision to be effective. But formal supervision was not necessarily helpful for everyone, with many finding informal debriefing with peers to be effective. However, this also required trust among colleagues. While the use of informal debriefing among colleagues was considered a sign of a healthy team, formal, structured debriefing was also common in some workplaces to support self-care. Use of humour and laughter was also an effective self-care strategy used in the workplace, with laughter often expressing a sense of acceptance, kindness and compassion for oneself rather than self-judgement during times when feelings of inadequacy arise.

Participants reported accessing a variety of professional supports as part of effective self-care practice. These ranged from Employee Assistance Programs and private counsellors to psychologists, general practitioners and other medical specialists. For doctors, it was considered especially helpful to seek objective medical advice from a general practitioner. Interestingly, choice of employer was a self-care consideration in terms of gauging organisational commitment to support staff with workplace self-care activities such as clinical supervision. Finally, effective self-care practice was described as a shared responsibility between palliative care professionals and the health services in which they practised. However, there can be a lack of clarity with regards to this shared responsibility.

### Theme 3: Barriers and enablers to self-care practice

Participants described the ongoing need to manage self-care barriers and enablers as part of maintaining self-care strategies in both personal and professional contexts.

#### Self-care barriers

Multiple impediments to self-care were identified in the workplace including busyness. For some, this workload was compounded by limited opportunity to take holidays from work. Workplace culture was also identified as problematic, where it was not conducive to self-care. In some workplace cultures there was a stigma associated with self-care, making it difficult for individuals to engage in self-care practice without feeling judged as being selfish. Bringing work home was described as a barrier to self-care, and related to workplace culture and expectations. Self-worth was also discussed as a common concern for effective self-care, where self-criticism and a lack of self-worth undermined self-care as an important priority. Finally, a lack of planning for self-care, or otherwise adopting a solely ad hoc approach was considered a barrier to effective self-care.

#### Self-care enablers

Several factors were described as facilitators of effective self-care. Recognising the importance of self-care was considered an important enabler by all. Some became conscious of this through previous experiences of illness or being unwell after having initially neglected self-care. Prioritising self-care was an important enabler which correlated with noticeable benefits. Adopting a preventative approach to self-care was important, whilst recognising that additional strategies may need to be implemented, as required, according to context. While formal self-care plans were used by some, for most participants it was more important to engage in reflection and self-assessment as part of an ongoing planning process, rather than have a static document.

Positive workplace cultures supportive of self-care were described as vital to effective self-care practice. Where a supportive culture was absent, the normalisation of self-care within workplaces was considered a key enabler, requiring leadership from the top-down to effect positive change towards a culture more supportive of self-care. One participant explained:

*Normalisation… the reason I bang on about [self-care] is because I think, yes, you do need to normalise it. I think the key thing is the ethos of the unit, and I think that’s set firstly through the medical head but then also the administrative or hierarchal structure [helps] - if it’s normalised and supported from the top then I think that flows down through the service… [from my observation] it is the leadership group of the team, and unfortunately that is still medical, who set the ethos of the unit. So, if you want to change the culture of the place, my approach would be to get the most senior consultants on board,* (Winston).

Leadership and positive role models were considered key enablers to effective self-care. This also related to the allocation of reasonable workloads. Other facilitators of effective self-care were more intrapersonal. These included having a positive outlook, self-awareness and positive emotions. Self-awareness was described as central to effective self-care practice. Gratitude and taking a positive perspective, even in the face of negative circumstances, enabled self-care. Self-compassion was considered essential to self-care, and relational to compassion for others - as expressed through patient care. One participant explained:

*Self-care is built on self-compassion. If your compassion does not include yourself, it is not complete; it extends to yourself and to your clients equally. And if you’re not doing that, then something’s not working* (Carmel).

Importantly, self-compassion did not necessarily come naturally, and in many cases had to be learned. For some, self-compassion was viewed as an emotion that became more apparent later in life, and when career achievement was less of a priority. Authenticity and courage were also described as self-care enablers. These encompassed self-advocacy and self-acceptance – in terms of being realistic about limitations; and being, in the words of one participant, *authentically human.* Lastly, reflecting on and having an appreciation of one’s own mortality was considered important and enabling for self-care practice.

## Discussion

This study explored the meaning and practice of self-care as described by palliative care nurses and doctors. These findings contribute new knowledge in several ways, with implications for clinical practice, research and education.

### A proactive and holistic approach to promoting personal health and wellbeing to support professional care of others

The holistic nature of self-care as revealed in this study is consistent with the discourse analysis conducted by Breiddal [21]. Findings from the present study extend this existing knowledge by providing new insight into the meaning of self-care, and also through further evidence of the relational context in which self-care is practised, as voiced by practitioners in the field. For palliative care professionals, self-care is not a selfish endeavour apathetic to the needs of others; rather, it is a proactive and relational practice cognisant of practitioners’ health and human needs, and motivated by the professional context of sustaining compassionate care in therapeutic relationship with patients and their families. This was especially evident in the words of one participant: …*if you don’t feed yourself, you’ve got nothing to give; much of what we do in palliative care is about human connectedness.* It also supports Kearney and colleagues’ [22] assertion that self-care is not a selfish luxury, but is instead essential to clinicians’ therapeutic relationship with patients.

### Personalised self-care strategies within professional and non-professional contexts

While most research has to date focused on strategies used to cope with occupational stressors [12]; these findings, situated in the broader context of self-care, reveal not only the variety of effective strategies employed, but also the challenges and complexities involved with maintaining effective self-care strategies in practice. The need for reflective practice to build self-awareness, as well as the management of multiple barriers and enablers to self-care practice, clearly demand ongoing attention from practitioners and palliative care services.

That mindfulness exercises were used spontaneously by participants in practice settings suggests that the benefits of formal mindfulness training initiatives extend beyond the training room and into the clinical milieu [23, 24]. Clinical supervision was effective for many, but not for others; and in many instances, it was not available at all. This seems to reflect, in part, a different attitude to clinical supervision within the nursing and medical disciplines; when compared to other disciplines such as social work, in which supervision has long been a cornerstone. As a social worker, Firth [25] explains that many nurses may feel threatened by supervision, whilst doctors have traditionally avoided it. Given the potential benefits to self-awareness and staff wellbeing, the provision of supervision should nonetheless be considered; perhaps with an emphasis on the restorative aspects of clinical supervision [25–27].

Formal and informal debriefing was consistently described as an effective self-care strategy, and thus should be encouraged. Similarly, laughter and the use of humour formed a fundamental part of self-care, and should be fostered as appropriate. Laughter has long been considered a coping strategy to manage stress in palliative care settings [28]; however, this finding extends a new context in terms of self-care behaviours to support health and wellbeing. Indeed, there is evidence to suggest not only psychological, but also physiological health benefits from laughter, including enhanced cardiac and immune function [29–31].

Establishing and maintaining effective boundaries within and outside of the workplace was an effective self-care strategy for participants in this study, as was work-life harmony. Whilst so-called work-life balance was discussed by some, and has also featured in other palliative care research into coping mechanisms [32]; this concept was incongruous to the experience of others. Overall, it was important to acknowledge that different life-domains require varying degrees of attention at any given time, and finding one’s individual harmony between personal and professional roles was thus a key strategy towards flourishing in life. This is consistent with McMillan and colleagues’ [33] definition of work-life harmony as ‘an individually pleasing, congruent arrangement of work and life roles that is interwoven into a single narrative of life’. It also corresponds with recent research findings that work-life interference, or conflict, is associated with higher levels of burnout in nurses and predicts intention to leave an organisation or the nursing profession [34]. Thus, work-life harmony is an important aspect of effective self-care. Given this finding, future self-care education might usefully incorporate this new emphasis on work-life harmony over the common parlance of ‘work-life balance’ which is ill-defined and otherwise problematic in practice for some [33].

Another interesting finding related to participants electing to work part-time as a self-care strategy. While only 42% of participants worked part-time in the present study, the majority of participants who had earlier completed a survey worked part-time. Given these and earlier research findings [32], working a part-time load appears to be a common self-care strategy for palliative care professionals. Indeed, one participant suggested that part-time roles should perhaps be encouraged in favour of a full-time load, given the emotionally demanding nature of palliative care. However, this would need to be weighed up by the individual in relation to feasibility of lower income and potentially limited opportunities for career advancement in roles where full-time work is required.

### Barriers and enablers to self-care practice

While positive workplace cultures were discussed as enablers of self-care, there were many who described their current workplace culture as a barrier to effective self-care, in that it was not supportive of self-care practice. This finding is alarming, yet not altogether surprising when taken in the context of self-care being highly stigmatised – as either selfish or weak – in some participants’ workplaces. Perhaps more concerning, is that this stigma may serve to not only impede effective self-care practice in the workplace; it could also discourage palliative care professionals from taking personal leave or seeking professional support when they become unwell. As described by Hill [35], a paediatric palliative care physician, showing vulnerability or seeking help is often viewed as a sign of weakness; and acknowledging one’s shared humanity and vulnerability through self-compassion is vital to self-care behaviours. Understanding factors that contribute to supportive workplace cultures and facilitate self-care is therefore essential. Some palliative care services in Australia might benefit from the experience of their counterparts in Canada and the United Kingdom, who have focused on leadership to foster workplace cultures of self-awareness, self-care, and staff support [36, 37].

Several enabling factors to self-care practice were identified in this study, both interpersonal and environmental. Authenticity, courage, and leadership were highlighted by participants. Being authentically human in acknowledging one’s own vulnerability; having the courage to challenge stigma or be assertive in saying ‘no’, when acquiescing to additional workload may compromise one’s own wellbeing; and leading by example in supporting and normalising self-care as an essential aspect of palliative care practice. Authenticity, courage, and leadership have been recognised as character strengths that can be measured and cultivated [38]. Development of these character strengths in palliative care teams should therefore be encouraged to assist in transforming any unsupportive workplace cultures.

In this study, positive emotions such as gratitude and self-compassion enabled self-care. This is consistent with a growing field of positive psychology research, in which positive emotions not only have a biological basis for physiological health benefits; but have also been shown to broaden repertoires of positive thoughts and actions which, in turn, help to build personal and social resources that lead to wellbeing and flourishing [39–43]. Whilst, in the context of psychological flexibility, negative emotions are not necessarily to be avoided [44]; awareness of, and capacity for the cultivation of, positive emotions should thus be fostered as part of self-care practice. This may serve to promote resilience and emotional intelligence both individually and across the palliative care team [45, 46].

That self-compassion was considered enabling to self-care, corresponds with findings from a recent correlational study [47] in which perceived self-care ability was significantly associated with increased self-compassion in palliative care nurses and doctors. Indeed, as highlighted by Vachon [48], self-compassion entails knowing and caring for oneself. The self-care barriers identified in the present study provide a valuable context which may also explain the low levels of self-care ability identified in some doctors and nurses from the previous study. Building from emerging evidence to support compassion-oriented training interventions in palliative care teams [24], future research should therefore investigate any causal relationship between these variables longitudinally. Potential studies could incorporate interventions that draw upon loving kindness meditation or other compassion training programs which have been shown to enhance compassion for self and others, and may therefore contribute positively to both self-care and compassionate care of others [49–53].

In other research [13, 54], palliative care professionals’ reported self-care practices have corresponded with physical, social, and inner domains of self-care. Importantly, findings from the present study underscore the imperative that strategies from these self-care domains are implemented and maintained in both personal and workplace settings. Findings from this study can thus inform the self-care education and training interventions recently called for [55], especially in relation to self-care planning, work-life harmony, and management of identified barriers and enablers to effective self-care practice. Educational resources might usefully draw upon this qualitative evidence previously lacking in the literature, to articulate and foster the meaning and practice of effective self-care in the palliative care workforce. For example, clarifying staff confusion about the shared responsibility for self-care practice – as identified in this study.

The issue of balance between individual and organisational responsibility is multi-faceted and requires careful consideration by palliative care services. Clearly, an organisation cannot practise self-care on behalf of its workforce; however, it can enable and enhance self-care through corporate leadership and a variety of structural supports to foster positive workplace cultures that are conducive to effective self-care practice [36, 37]. At the same time, individual practitioners carry a personal responsibility for self-care to maintain their health and capacity for professional practice. This was highlighted by one participant, who stated: *It’s the responsibility of every team member to look after themselves, but having management or organisational strategies in place to support someone doing self-care is incredibly important… it’s a dual process.* This collaborative approach to promoting health and wellbeing in workplace contexts is reflected in the World Health Organisation’s (WHO) [56] Healthy Workplace Framework.

While individual responsibility relates to implementing and maintaining self-care strategies, organisational responsibility is thus oriented towards supporting staff in effective self-care practice to promote health and wellbeing. This support represents an investment, with a host of potential organisational benefits including increased patient and family satisfaction, increased staff retention and reduced absenteeism, improved staff morale and job satisfaction [27]. Conflict between colleagues can be a common source of staff stress, and is an important workplace concern where all parties must take some responsibility. While the degree of responsibility will vary according to context, the use of employee assistance programs and adoption of the WHO Healthy Workplace Framework can provide support and guidance in this area.

Clarity may also be lacking with regards to shared responsibility for self-care practice where clinicians experience chronic illness and or disability. Health services, as institutions, have the potential to promote health and wellbeing not only for health care consumers; but also for health care professionals [57]. Indeed, some argue that hospitals should serve as exemplars of healthy workplaces [58]. Given this context, a collaborative approach encompassing individual self-management and organisational support would be consistent with the WHO Healthy Workplace Framework, which recommends that workplaces be supportive of employees living with chronic disease and disability [56]. Palliative care services might usefully draw upon this or similar approaches.

Given the highly personalised nature of self-care, palliative care services should also consider ways in which a variety of self-care strategies can be supported. For example, providing opportunities for both informal debriefing and formal clinical supervision – depending on individual preference; as well as scope for the supported development of individual self-care plans for those who feel they would benefit from them.

A novel finding from this study was the concept of team-care to promote a healthy team. As an encouraging sign of positive workplace cultures, this highlights an additional dimension to the relational context of self-care practice, whilst underscoring the importance of supporting interdisciplinary teamwork as an integral part of the philosophy of palliative care. It also contributes to the literature on positive relationships and workplace wellbeing in the context of self-care and positive health [59]. Taken together, the practice of team-care as an antecedent to a healthy team in palliative care represents a potential avenue of qualitative inquiry for future research. This would be enhanced with the inclusion of participant observation and patient-reported outcomes on any perceived benefits to the quality of care provided.

### Limitations

Limitations to this study should be noted. Socio-cultural considerations were not represented in the demographic data collected or subsequent analysis. Whilst, to our knowledge, any significant impact of culture on self-care has not featured in the literature to date, we acknowledge that palliative care professionals from culturally and linguistically diverse backgrounds may understand and approach self-care practice in ways other than as described in this study sample. Additionally, the sample was somewhat limited in terms of participants’ geographical location. While the study recruited participants from metropolitan and inner or outer (rural) regional locations of nearly all Australian States and Territories, remote area locations were not represented. The meaning and practice of self-care may have unique characteristics in remote area practice, thus transferability of findings from this study should be gauged by remote area practitioners. Despite these limitations, which may be addressed in the future by discrete population-specific studies, this research has generated new knowledge in line with the study aim.

## Conclusions

The findings of this study reveal a context and complexity of effective self-care practice previously lacking in the literature. Taken together, the findings of this research provide new insight to support palliative care practice and education. Self-care is a proactive and personalised approach to the promotion of health and wellbeing through a variety of strategies, in both personal and professional settings, to support capacity for compassionate care of patients and their families. Importantly, it is a shared responsibility between palliative care professionals and the palliative care services in which they work, with staff support and positive workplace cultures required to manage various barriers and enablers to effective self-care practice. This research adds an important qualitative perspective and serves to advance knowledge of both the context and effective practice of self-care in the palliative care workforce.
